# How to link organizational resilience to transformational entrepreneurship behavior as theoretical framework gap – A systematic literature review

**DOI:** 10.12688/f1000research.133459.1

**Published:** 2023-06-28

**Authors:** Michael Gunawan, Budi Soetjipto, Lily Sudhartio

**Affiliations:** 1 Faculty of Economics & Business, University of Indonesia, Depok, Indonesia

**Keywords:** Organizational resilience, transformational entrepreneurship behavior, socio-economic growth, environmental sustainability, community development

## Abstract

**Background:** Numerous enterprises face great challenges during uncertain economic conditions. This is particularly true for micro, small, and medium-scale companies, which are slumped against disruption from the COVID-19 pandemic—owing to lockdowns, decreased demand, and a disrupted supply chain. This has impacted the economy worldwide but also the social community and the environment that forms its ecosystem. Organizational resilience allows for socio-economic growth and enterprises to build environmental sustainability and balanced community development. Therefore, the behavior of companies must be transformed in building entrepreneurship to encourage socio-economic growth.

**Methods:** We conducted an advanced search on Business Source Premier, ABI/INFORM (ProQuest), Emerald Insight, and Web of Science database between March and June 2022. We screened the bibliographies of the articles from the database search using a set of inclusion criteria such as studies with quantitative design with unit analysis population sample based microfinance institutions and cooperative-based microfinance institutions, MSEs, and MSMEs as well as other industries such as travel agents, property, restaurants, food and beverages, manufacturing and plantations; exploration of transformational entrepreneurship behavior and organizational resilience determinant factors and conceptual manuscripts written in English; and published between 2005 till 2019 for transformational entrepreneurship and 1997 till 2000 for organizational resilience.

**Results:** This study reviewed 22 articles focusing on the role and influence of organizational resilience on transformational entrepreneurship behavior from a lens that posits the importance of organizational resilience in the face of uncertain economic turbulence. The orientation of entrepreneurship behavior has been neglected in research so far. This systematic literature review study reveals important research gaps, such as the influence of organizational resilience in promoting the development of transformational entrepreneurship behavior and the determinants that build organizational resilience.

**Conclusions:** Research related to a company’s transformational entrepreneurship behavior offers a broad area of ​​scientific research; hence, encouraging further investigation is necessary.

## Introduction

Economic growth has suffered dramatically owing to the ongoing COVID-19 pandemic, which has shown no end as of yet. The situation continues impacting socio-economic development, environmental sustainability, and people’s welfare. Meanwhile, the epidemic situation, which resulted in a decline in demand and purchasing power, prompted companies to be proactive, innovate, and take risks to recover and improve their economic performance ( Covin & Slevin, 1991; Miller, 1983; Scherer et al., 1989). The efforts exerted, thus far, to achieve economic growth have fallen short of achieving sustainable growth. Therefore, transformational behavior from organizational leaders, which includes innovative, proactive, and risk-taking behavior, is needed to achieve socio-economic growth, where social development can include environmental sustainability and community development in an optimal and balanced manner ( Maas et al., 2019; Maas & Jones, 2017). Similarly, Roth and DiBella (2015) argued for the importance of encouragement from top management to foster transformational behavior within a company and certain skills, such as how to read the current environmental conditions, be able to absorb new knowledge from existing situations and conditions, and influence the transformation at various levels in the company to implement a new approach.

This is a conceptual paper produced through an in-depth literature review. It attempts to answer the following questions:

1. Transformational entrepreneurship behavior is so important to achieve socio-economic growth that it includes environmental sustainability and community development in a balanced way; then, why is this entrepreneurship behavior lacking?
2. What determinants shape transformational entrepreneurship behavior toward socio-economic growth?
3. What are the determinants that shape and build organizational resilience?

**Figure 1.**
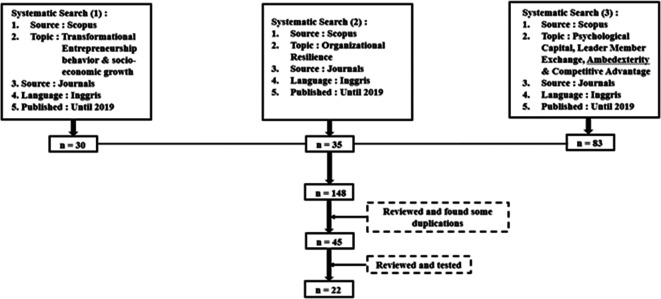
Process of searching and selecting systematically.

This conceptual study contributes to the literature on transformational entrepreneurship behavior ( Table 1) and the impact of organizational resilience ( Table 2). The following contributions have resulted in a research framework ( Figure 1) and provided a pathway for further research. This study provides a novel opportunity to take a closer look at the influence of organizational resilience and other determinants on transformational entrepreneurship behavior. To the best of the authors’ knowledge, this study is the first in-depth and systematic review of the literature to consider the transformational entrepreneurship behavior essential for a company to achieve sustainable goals. Second, this study builds a logical theoretical framework model to connect several determinant factors in building organizational resilience. The conceptual framework was formed through a literature review and theoretical gaps.

**Table 1.** Transformational Entrepreneurship - Research Streams and supporting theories.

Research streams	The supporting theories	Elucidation
Social-economy growth	Transformational entrepreneurship	Transformational entrepreneurship (TE) is oriented holistically and heuristically in terms of promoting entrepreneurship and combining individuals and subsystems, namely communities and institutions/companies, to interact and collaborate to create synergy in the form of a framework that positively impacts obtaining opportunities to be utilized at the local or regional level ( Maas et al., 2019). Indubitably, the transformation of entrepreneurship requires a systematic and holistic approach, precipitating transformational change in encouraging the growth of socio-economic development. Entrepreneurial transformation encourages the growth of socio-economic development, including an optimal balance between economic development and social growth, including environmental sustainability & community development in a balanced and simultaneous way ( Maas & Jones, 2017). TE was found to contribute largely to socio-economic and long-term impacts, including concurrent community development ( Schoar, 2010). Meanwhile, a combination of technology-intensive and social growth-oriented entrepreneurship is also needed to overcome the current stagnation in terms of global socio-economic growth. Therefore, the TE domain focuses on research to find a better way to overcome the current challenges and constraints to create a better future through a comprehensive systemic and holistic approach to achieve future socio-economic growth ( Marmer, 2012). In the end, transformational entrepreneurs are expected to be able to create innovative solutions to the world's biggest problems that are measurable, sustainable, and systematic to achieve balanced and simultaneous socio-economic growth ( Marmer, 2012).
Transformational behavior	Transformational entrepreneurship behavior	Entrepreneurship behavior has a transformational impact on their communities regarding social, economic, environmental, and community development performance ( Maas & Jones, 2017). Therefore, effective entrepreneurship behavior is needed to achieve transformational entrepreneurship by engaging in transformational proactive behavior, transformational innovative behavior, and transformational risk-taking behavior to achieve socio-economic growth. Furthermore, entrepreneurship behavior also includes the skills, competencies, knowledge, and characteristics required to foster the growth of transformational companies ( Ratten & Jones, 2018).

**Table 2.** Psychological capital, leader member exchange, ambidexterity and competitive advantage strategy - Research streams and supporting theories.

Research streams	The supporting theories	Elucidation
Hope, optimism, resilience, and efficacy	Psychological capital	According to Chiang and Hsieh (2012), business and market competition is dynamic and changing, following the strategic environment that changes extremely quickly with changes in high market demand, the organization must have the ability to innovate as a form of creativity to ensure that it has a high competitive advantage. These organizations can provide quality services with competitive advantages and can meet diverse customer needs ( Chang & Teng, 2017; Hon, 2012; Hon & Lui, 2016; Li & Hsu, 2016). However, the creativity of employees in encouraging the development of organizational innovation will run well with full and positive support from their superiors. Likewise, Chen and Wu (2017) and Patiar and Wang (2016), through recent empirical studies, posited it has been proven that good leadership has a positive impact that greatly affects employee behavior in an organization. Positive leadership can increase morale, motivate employees, and affect employee commitment to create a high performance for the organization and encourage higher service quality improvements overall ( Testa & Sipe, 2012; Uen et al., 2012). On the contrary, if the leader cannot encourage a positive mentality from their employees, motivate, and encourage behavior change through empowerment to improve the organization's quality, then, of course, it will result in a decrease in overall organizational performance. Several empirical studies have demonstrated that a positive correlation exists between various types of leadership and psychological factors and employee behavior. A perceptual attitude (stance) combines a sense of reality, wisdom, positive perception, and a high commitment to integrity. Therefore, forming a perception that can be widely and comprehensively accepted within the organization as a fundamental foundation is necessary to encourage the growth of organizational progress that has desirable resilience capabilities ( Kantur & İşeri-Say, 2012). Meanwhile, Hind, Frost, and Rowley (1996) stated that understanding reality about the perception of self-image, which is in line with the real situation, is an element of resilience at the organizational level. Perception of the real reality is very important so that the weaknesses and strengths of the organization can be understood properly. Organizations with strong resilience consist of leaders and employees with positive perceptions, high beliefs, and a good attitude of optimism that shape the organization's character. However, it is necessary to be aware of the vulnerabilities and weaknesses of the organization so that anticipatory steps are needed to take appropriate action ( Kantur & İşeri-Say, 2012). Psychological capital (PsyCap) is highly decisive and, as a causal factor, determines organizational resilience to support the success of transformational entrepreneurship to produce increased employee performance.
Loyalty, affect, contribution, and respect	Leader-member exchange multdimension	Engagement with high commitment and empowerment of employees in behaving and acting is a very important foundation. Employees must demonstrate appropriate behavior and attitudes in responding to difficult, chaotic, or even uncertain environments. In times of high pressure and uncertain turbulence, organizations can adapt appropriately to various situations and conditions to form effective organizational resilience ( Mallak, 1998). Empowering employees through the provision of authority is proven to have increased capability in the decision-making process. It is proven to produce innovative and creative solutions. According to Mallak (1998), employee empowerment is one essential element of organizational resilience in building high self-efficacy and confidence. Resilience at the individual level is largely determined by the full engagement of employees to bring the organization to adapt to volatile situations and conditions that result in a changing environment ( Doe, 1994). The quality of the exchange of reciprocal relationships between leaders and employees in the form of mutual trust, mutual openness, and mutual support in an organization determines the quality of an organization and forms a good organizational leadership character. Meanwhile, distance is very meaningful for followers and leaders or subordinate manager relationships because the level of closeness in the relationship between leaders and employees/individuals they lead greatly affects "the behavior of followers/individuals under them to produce a performance that is important for organizational effectiveness ( Graen et al., 1982; Rothaus et al., 1965)”. The relationship between leaders and followers is highly dependent on power distance; power distance also affects commitment to full involvement and also empowerment in the form of authority to followers ( Anand et al., 2011; Erdogan & Liden, 2002; Liden et al., 2016). The shorter the power distance between leaders and employees, the better the quality of reciprocal relationships in the form of mutual trust, mutual love, mutual influence, and communication with each other ( Liden & Graen, 1980).
Exploration and exploitation capabilities	Ambidexterity	The essence of exploration is experimenting with innovations that are new alternatives. The process is long, and the return is uncertain, far and often negative, from the investment that has been expended. Meanwhile, the core of exploitation is the improvement and renewal of the organization's ability to continuously explore existing opportunities and develop better ones that positively impact the organization ( March, 1991). Organizations must maintain an appropriate combination of exploration and exploitation—a balance between the organization's ability to exploit and explore existing and new opportunities to produce optimal company performance ( Cao et al., 2009; Lubatkin et al., 2006). According to O'Reilly and Tushman (2008), the necessity for the simultaneous execution of exploration and exploitation is purposed to achieve a balance that results in the survival and success of the company. Hence, combining exploration and exploitation is highly important to achieve optimal long-term performance and success ( Benner & Tushman, 2003; Gibson & Birkinshaw, 2004; Lubatkin et al., 2006; Tamayo-Torres et al., 2017). Furthermore, Mallak (1998) posited that organizations can withstand sudden and unpredictable crises and disasters because they have qualified human resources to form high organizational resilience. While relational resources are important as sources of contextual integrity that contribute to organizational resilience, other tangible and intangible resources are required to increase resilience. This is coherent with organizational ambidexterity, which refers to the organization's ability to explore and exploit to compete in mature technologies and markets where corporate values, including competence, technological mastery, and organizational agility to face new challenges, are required ( Lubatkin et al., 2006; O’Reilly & Tushman, 2013). Ambidexterity is positively related to sales growth ( Caspin-Wagner et al., 2012; Geerts et al., 2010; Han & Celly, 2008; Iyer et al., 2006), innovation ( Eisenhardt & Tabrizi, 1995; Katila & Ahuja, 2002; McGrath, 2001; Tushman et al., 2010), and corporate viability ( Hill & Birkinshaw, 2014; Kauppila, 2010; Laplume & Dass, 2012; Yu & Khessina, 2012)”. Organizations with effective resilience capabilities are invariably prepared to face disasters, volatile situations, and conditions, and massive crises that may occur in the future and are considered to have strong organizational resilience. The strategic value of organizational capacity needs to be followed up with a proactive plan for strategic action in which the behavior is responsive, proactive, flexible, and has high creativity to build a competitive advantage to achieve organizational goals that innovate ( Duchek, 2020; Kantur & İşeri-Say, 2012; Wenzel et al., 2021).
Differentiation, neutralization, exploitation, and cost leadership advantage	Competitive advantage strategy	Every company must have a competitive advantage strategy to achieve the company's goals and objectives ( Ehie & Muogboh, 2016; Kharub & Sharma, 2016, 2017; Olhager & Feldmann, 2018). An in-depth study of the bibliography shows that there are 4 (four) categories that reduce the concept of a competitive advantage strategy, namely (1) how the elaboration of the concept of strategy creates a competitive advantage ( Kathuria et al., 2007; Porter, 1985, 2000; Skinner, 1969, 1985); (2) the emphasis of the definition of competitive advantage strategy ( Dangayach & Deshmukh, 2006; Ward & Duray, 2000); (3) how to build a correlation between competitive advantage strategy and company performance ( Amoako-Gyampah & Acquaah, 2008; Olhager & Feldmann, 2018), and (4) encouraging the capability development to support the company's competitive advantage strategy ( Jarzabkowski et al., 2016; Sharma & Kharub, 2014). A company's success in achieving the goals and objectives that have been determined is strongly influenced by the competitive advantage strategy ( Korutaro Nkundabanyanga et al., 2014). Porter designed a framework for building competitive advantage, namely cost advantage and differentiating elements (differentiation), to obtain better performance ( Kharub & Sharma, 2016, 2017). Organizations implement a competitive advantage strategy supported by effective and qualified human resources ( Kathuria et al., 2007; Song et al., 2018). Organizations demand to build a competitive advantage to develop a business that is oriented to focus on its target market. Meanwhile, in business, a competitive advantage category is needed, according to Barney (1991), VRIN: Valuable, Rare, Inimitable, and Non-Substitution. If this competitive advantage is built properly, then the continuity of the organization or company will run in the long term ( Jacobsen, 1988; Porter, 1985). Furthermore, according to Porter (1985, 1989), Barney (1991, 1995), Christensen (2006) **,** Sigalas, Economous, and Georgopoulos (2013) **,** Stonehouse and Snowdon (2007), and Nkundabanyanga, Akankunda, Nalukenge and Tusiime (2014) competitive advantage strategy comprises of essential dimensions as follows: Distinguishing Advantage (differentiation), Cost Leadership Advantage, Market Exploitation Advantage, Threat Neutralization Capability, and Cost Reduction Capability.

The remainder of this paper is structured as follows: (1) We describe the classification and provide a thorough explanation of organizational and transformational entrepreneurial behaviors. (2) We review and briefly describe an approach to descriptively review and analyze a collection of literature. (3) We systematically design the conceptual framework referring to the previous step as the basis. (4) The main findings obtained are then discussed, fostering a potential pathway for a detailed description for further research. This paper also describes its limitations; the final section presents the conclusions.

### Transformational entrepreneurship behavior

Companies need leaders who foster transformation to survive in a turbulent environment and face uncertainty. Leaders who do this successfully have high integrity and motivate and encourage the efforts of their employees to achieve organizational goals and targets to improve company performance ( Lafley, 2009). Therefore, entrepreneurship behavior, which includes innovative, proactive, and risk-taking behaviors, is essential ( Covin & Slevin, 1991; Miller & Friesen, 1982). Meanwhile, Bass (1985) and Howell and Avolio (1993) held that transformational leaders encourage the development of innovation in their organizations. This is also supported by Crant (2000) and Deluga (1998), who found that transformational leadership encompasses proactive, innovative, and risk-taking behavior. In other words, transformational leadership has been recognized to be connected to entrepreneurship.

Therefore, there is a need for renewed thinking to stimulate entrepreneurship to support socio-economic growth. In this context, ‘transformational entrepreneurship’ refers to a holistic and heuristic orientation in terms of promoting entrepreneurship and bringing together individuals and other subsystems, such as communities and institutions that interact and collaborate to create a positive framework within which opportunities can be leveraged that extend beyond the local area ( Maas et al., 2019).

Then, how important is the role of entrepreneurship in encouraging sustainable socio-economic growth to ensure that organizations may realize the right capabilities, capacities, and policies, including the ecosystem that supports them, to change or maintain a progressive socio-economic landscape—including community development in Indonesia through balanced optimal growth? Entrepreneurship can potentially precipitate a measure of success in dealing with volatile situations and conditions with high uncertainty. In times of strife, a company must not merely seek survival but also the ability to grow sustainably. Therefore, companies do not solely need economic returns (short-term) but also social impacts (long-term), such that the company’s success contributes to societal welfare ( Maas et al., 2019; Ratten & Jones, 2018; Xu & Maas, 2019).

Wealthy people are economically capable of sustaining a company’s progress, thus allowing the company to grow sustainably. Entrepreneurship also seeks to make a social impact by developing socially-oriented entrepreneurship. However, social entrepreneurship focuses on “community work, voluntary and public organizations, and private enterprises working for purely social purposes rather than for profit” ( Shaw & Carter, 2007). Solutions tend to rarely be formulated with consideration for economic scalability for sustainable socio-economic growth ( Marmer, 2012). On the contrary, we need entrepreneurship that can transform profit-oriented companies to include a social growth perspective in their goals.

Thus, a new approach is needed in socio-economic development through a systemic and holistic process to accommodate the needs of individuals economically and socially ( Maas et al., 2019). Without a new paradigm in the form of new approaches, such as transformational entrepreneurship, the potential for socio-economic development will remain limited and only benefit a small number of individuals, businesses, and organizations. As it is necessary to establish principles related to transformational entrepreneurship and the ecosystems that support it, practical cases are provided to illustrate the global concept of transformational entrepreneurship ( Maas et al., 2019). Therefore, transformational entrepreneurship behavior focuses on dynamically stimulating socio-economic development to achieve balanced optimal growth ( Maas et al., 2019; Ratten & Jones, 2018).

To address this need, the concept of transformational entrepreneurship is introduced. Transformational entrepreneurship encourages the advancement of entrepreneurship by bringing together individuals, communities, and institutions that interact and collaborate to take advantage of existing opportunities and reach a wider scale to promote optimal and balanced socio-economic growth ( Maas et al., 2019; Ratten & Jones, 2018; Xu & Maas, 2019). Additionally, transformational entrepreneurship encourages entrepreneurial activities that bring about meaningful changes in markets and industries, sociocultural life, and environmental and community development ( Marmer, 2012).

According to Maas et al. (2019), balanced socio-economic growth must be achieved through activities that encourage the development of entrepreneurship and change in society at large, which positively impacts socio-economic growth, including community development. Therefore, to overcome current phenomena such as poverty, low welfare, inadequate health, unemployment, and negative business growth, a transformation is needed by supporting entrepreneurship as part of a comprehensive system, namely a system consisting of community, public, private, environmental, and natural resource management that provides benefits.

Research has demonstrated that company performance can be improved if top leadership exhibits entrepreneurship behavior ( Andole & Matsui, 2021; Dess & Lumpkin, 2005; Llanos-Contreras et al., 2020; Majid & Koe, 2012; Scherer et al., 1989). However, entrepreneurship behavior is more focused on obtaining economic benefits, even though what is needed is to build a sustainable company ( Aldrich & Zimmer, 1986; Chell & Baines, 2000; Dubini & Aldrich, 1991). A sustainable company can bring the company to achieve long-term business goals through its processes and actions from time to time by incorporating economic, social, and environmental aspects into its business strategy ( Baumgartner & Rauter, 2017; Haugh & Talwar, 2010).

Consequently, entrepreneurship behavior is no longer adequate; therefore, Maas *et al.* (2019) introduced transformational entrepreneurship behavior (TEB), which includes transformational proactive behavior, transformational innovative behavior, and transformational risk-taking behavior. The aim is enhancing people’s welfare and prosperity, thus increasing their purchasing power and consumption.

Several previous studies have explained the factors driving TEB, including Xu and Maas (2019), Roth and DiBella (2015), and Maas *et al.* (2019), wherein the TEB is formed in a dynamic context. Meanwhile, entrepreneurship behavior, especially for transformation, must be action-oriented and, indubitably, require the capability to realize these actions. Therefore, it takes the company’s capabilities to realize the TEB. Moreover, Roth and DiBella (2015) posited that five capabilities are needed to enable transformational change: company awareness about the situation and market conditions and the environment in which the company is located related to competitiveness, ability to innovate, the balance of encouragement from top management, and absorption of new knowledge about the current situation.

These capabilities not only adapt to change but also recover when hit by change ( Duchek, 2020; Garud et al., 2007; Kantur & İşeri-Say, 2012; Wenzel et al., 2021). These three capabilities form the core of organizational resilience ( Duchek, 2020). Korber and Naughton (2018) held that OR is a determinant of entrepreneurship behavior and intentions; thus, OR is often manifested in self-efficacy or optimism.

According to Duchek (2020), organizations that prepare themselves to anticipate a crisis (self-preparation capability) can act proactively, creatively, and innovatively by preparing human resources, organizational resources, and infrastructure to build systems to deal with potential crises. Further, Duchek (2020) posited that when a crisis occurs, companies that are already prepared can respond to the crisis and then absorb and adapt to the changes caused by it. Proactiveness, creativity, and innovation are crucial components of entrepreneurship behavior, including TEB, whereas self-prepared organizations are more resilient when experiencing change. Therefore, arguably, OR may affect TEB.

Other determinant factors include how the organization can take advantage of existing opportunities and customers and seek new opportunities—new customers. On the one hand, the organization can exploit existing opportunities; on the other hand, it can seek new opportunities with a balanced creative and innovative approach ( Benner & Tushman, 2003; Cao et al., 2009; Gibson & Birkinshaw, 2004; Lubatkin et al., 2006; O’Reilly & Tushman, 2008, 2013; Tamayo-Torres et al., 2017).

Companies—in maintaining and retaining existing customers, as well as opportunities to find new customers—must have a strategy of having a competitive advantage because the company also has competitors in the region where it is located. Therefore, companies need to have competitive and unique products and services superior to those of similar competitors to create a competitive advantage ( Barney, 1991, 1995; Flamholtz & Randle, 2012; Porter, 1989, 1996; Stonehouse & Snowdon, 2007).

### Organizational resilience

Considering that the occurrence of turbulence, situations, and conditions categorized as social and environmental conditions are currently experiencing an uncertain and unpredictable situations, efforts are needed to increase resilience to include organizations, individuals, regions, and social communities ( Kantur & İşeri-Say, 2012). Therefore, resilience encompasses a broad range of industries and scientific disciplines ( Gunderson & Holling, 2002; Werner & Smith, 1977).

Hence, in organizational studies, an understanding of organizational resilience is the ability of an organization or a region to apply crisis management to face disasters, so organizations need high and even the highest reliability to overcome problems that occur suddenly and cause serious disruption. ( Sutcliffe & Vogus, 2003; Weick et al., 1999; Weick & Sutcliffe, 2001). Horne and Orr (1997) defined resilience as “a fundamental quality in terms of the capacity and capability of individuals, groups, organizations, and the system as a whole to respond effectively and productively” to large and sudden changes that are massive so that they disrupt or damage the existing system and can cope within a short period. Meanwhile, Mallak (1998) stated that resilience is necessary under sudden shocks such as natural disasters or terrorist attacks and is also relevant for an organization, a region, and an individual faced with sustainable socio-business and environmental transformation.

Similarly, Robb (2000) posited that organizations with strong resilience can provide outstanding performance following current organizational goals and continue to innovate and adapt to changing market conditions and technology. Overall, practitioners believe that organizations with strong resilience automatically can successfully avoid disturbances while also coping with turbulence disturbances that occur effectively.

Accordingly, transformational entrepreneurship behavior that brings innovation faces the challenge of implementing it in companies at various scales, as well as the difficult challenge of becoming a mission and transformational behavior in MSMEs, as well as how micro- and small-scale financial institutions foster the growth of MSME companies to achieve socio-economic sustainability and community development ( Maas et al., 2019; Xu & Maas, 2019). The literature has largely ignored the determinants of transformational entrepreneurship behavior. Entrepreneurship behavior still dominates contemporary companies—namely, the current flow of research that is merely oriented toward economic growth ( Guiso et al., 2021; Zahra, 2021) and, thus, largely ignores sustainable growth—both environmental and socio-economic ( Maas et al., 2019; Xu & Maas, 2019). These facts are extremely important, considering that the nature of transformational entrepreneurship behavior differs from entrepreneurship behavior, where transformational entrepreneurship behavior is frequently associated with socio-economic growth ( Maas et al., 2019).

### Transformational entrepreneurship in organizations

Facing conditions and a volatile and uncertain environment, companies need to have capable leaders with high integrity to ensure that they can transform the company to achieve its goals and objectives in creating socio-economic growth, including community development ( Maas et al., 2019).

In conducting transformational entrepreneurship, companies are not only innovative, proactive, and risk-taking behavior ( Bass, 1985; Covin & Slevin, 1991; Crant, 2000; Deluga, 1998; Howell & Avolio, 1993; D. Miller & Friesen, 1982) for economic purposes only, but sustainable as well—by possessing transformational proactive behavior, transformational innovative behavior, and transformational risk-taking behavior to achieve socio-economic growth comprising environmental sustainability and community development interests simultaneously and in optimal balance ( Maas et al., 2019).

However, in reality, extant research still discusses entrepreneurship that focuses on the economic aspect ( Guiso et al., 2021; Zahra, 2021), elucidating how entrepreneurship brings organizations or companies to grow and develop to achieve competitive advantage and economic advantage. These studies do not explore the fact that the competitive advantages and economic benefits as explicated may only be relatively short-term and unsustainable, whereas producing competitive advantages and economic benefits that are relatively long-term and sustainable requires the support of the surrounding environment sustainability in the form of community development that is concurrently related with socio-economic performance ( Maas et al., 2019).

Thus, widely discussed in various journals, entrepreneurship is likely to exhibit a limited impact because the solutions are rarely designed with scalability and long-term sustainable economic and social development ( Maas et al., 2019; Xu & Maas, 2019). Therefore, understanding entrepreneurship from a social perspective and the development of the environment and society from a balanced and simultaneously economic perspective is necessary.

The second challenge is the realization of transformational entrepreneurship. Considering the wide scope of transformational entrepreneurship behavior, it is likely that only strong and resilient organizations can support leaders to do so. A strong and resilient organization is one with high organizational resilience. Meanwhile, according to Doe (1994), Horne (1997), Horne and Orr (1997), Mallak (1998, 1999), and Warner and Pyle (1997), organizations with effective resilience capabilities can face and survive in turbulent, chaotic, and uncertain situations. Organizations that have high resilience are also prepared to face even the worst situations and respond “appropriately and quickly” ( Sullivan-Taylor & Wilson, 2009; Wilson et al., 2010).

## Methods

The author conducted a literature review to study and develop a body of knowledge based on the results of journal-journal research as a guide for future research agendas. According to Tranfield et al. (2003), an in-depth study of existing journals should be conducted through systematic reviews, which help find research gaps and determine the future scope of investigation in the field.

### Data collection

From March to June 2022, a search was conducted using the Business Source Premier, ABI/INFORM (ProQuest), Emerald Insight, and Web of Science. From the database search, we screened the bibliographies of articles according to a set of inclusion criteria outlined below, including studies with quantitative designs involving unit analyses with population samples and cooperatives. It explores transformational entrepreneurship behavior and organizational resilience determinant factors as well as conceptual manuscripts written in English, including MSEs and MSMEs, as well as other industries such as travel agents, property, restaurants, food and beverage, manufacturing, and plantations. Articles on transformational entrepreneurship published between 2005 and 2019 and organizational resilience published between 1997 and 2000 were selected for this review. ProQuest, Emerald Insight, and Web of Science databases (via e-Library of the University of Indonesia) were used to search Google Scholar, Business Source Premier, and Business Source Premier.

The authors researched related journals through the string “AND” and “OR” ( Tremml, 2019; Whitten et al., 2007), which are “Transformational Entrepreneurship” AND “Socio-Economic Growth,” “Organizational Resilience” AND “Competitive Advantage.” Then “Transformational Entrepreneurship Behavior” AND “Innovation, Risk-Taking and Proactive,” thus “Organizational Resilience” AND “Leader-Member Exchange, Psychological Capital” AND “Competitive Advantage,” “Transformational Entrepreneurship (Behavior)” AND “Competitive Advantage.” Moreover, “Transformational Entrepreneurship Behavior AND Transformational Entrepreneurship” were searched for the latest journals in the 2010-2020 period and Organizational Resilience for the period 1997-2020, which became a theoretical foundation like “transformational entrepreneurship (behavior) theory” ( Maas et al., 2019; Maas & Jones, 2017; Marmer, 2012; Xu & Maas, 2019) and “organizational resilience” ( Duchek, 2020; Horne, 1997; Horne & Orr, 1997; Kantur & İşeri-Say, 2012; Välikangas & Romme, 2012). These collected and reviewed journals can be classified as validated such that they significantly impact the knowledge derived and research conducted ( Keupp et al., 2012; Podsakoff et al., 2005).

*Transformational entrepreneurship*

Inclusion criteria:

- Quantitative research and literature review papers
- Published in between 2005-2019
- Accessible without paying

Exclusion criteria:

- Articles published before 2005 and after 2019

*Organizational resilience:*

Inclusion criteria:

- Quantitative research and literature review papers
- Published in between 1997-2020
- Accessible without paying

Exclusion criteria:

- Articles published before 1997 and after 2020.
- Both quantitative research and literature review paper.

### Data analysis

Following Tremml (2019) and as recommended by Booth et al. (2022), a careful selection process produced 148 manuscripts, which were analyzed in full depth. Based on the inclusion and exclusion criteria, 45 articles were shortlisted for the review, 28 articles for organizational resilience, and 17 articles for transformational entrepreneurship. This is in accordance with Tremml, who argued that it is necessary to examine a reference in more detail as a technical search to ensure that it lends a meaningful addition to reference checking as an additional search technique ( Booth et al., 2022). Ultimately, 22 studies were included in this review. Figure 1 presents the systematic search and selection process. The articles obtained were then carefully examined and studied related to transformational entrepreneurial behavior strategies, organizational resilience, psychological capital, leader-member exchange, ambidexterity, and competitive advantage, and can be applied in all industries, including MSMEs, microfinance institutions, and multi-finance cooperatives.

During the process of a systematic literature review, the authors were aware of potential sources of bias and took steps to minimize their impact. This involved using strict inclusion and exclusion criteria to identify relevant studies as well as assessing the quality and validity of the research methods employed in each study. We considered the potential impact of bias on the conclusions drawn from the research and reported any limitations or biases that may have influenced the findings. To link organizational resilience to transformational entrepreneurship behavior, it is essential to identify and address any potential sources of bias that may impact the theoretical framework of the research. This involved a thorough and critical review of the existing literature as well as a clear understanding of the underlying concepts and theories involved. By carefully considering the potential for bias at each stage of the research process, we developed a robust and reliable theoretical framework that can guide future research in this area. This literature review assesses the risk of bias by considering the tendency of primary studies and research published in proceedings and journals to be examined with only a significant effect; those that are not significant are not included.

Transformational Entrepreneurship (behavior measured for research) is a relatively new topic; therefore, the timeframe for publishing a journal is shorter and more limited than organizational resilience (much longer and has become a significant issue in the 1990s). Transformational entrepreneurship (behavior) departs from entrepreneurship (behavior/orientation) and social entrepreneurship; The review started in 2005 through Mel et al. regarding entrepreneurs possessing qualities of high willingness to take risks and high managerial and financial literacy to innovate. Miller and Collier (2010) attempted to develop transformational entrepreneurship following Schoar (2010). Furthermore, Marmer (2012), through the Harvard Business Review, emphasized transformational entrepreneurship (TE) in four quadrants, which is the future of organizations. Companies must develop TE to achieve socio-economic growth and environmental and community development in an optimal and balanced manner. Jones, Lockyer, and Maas and the Jones and Maas couple developed TE continuously through thoughts and concepts, as well as case and empirical studies from 2019 until now.

While the topic of organizational resilience has been around since the 1990s, resilience also includes regional/city resilience to natural disasters, earthquakes, agency hurricanes, and organizational/company resilience to sudden economic turbulence. Therefore, the period for organizational resilience in this manuscript review began earlier, from 1997 to 2020, and is still ongoing today (mainly when the Covid-19 pandemic occurred).

We conceptualize resilience as a meta-capability, including the determinants and ecosystems that shape it, and break down the construct into its parts or the dimensions that make it up.

Our analysis is based on the data and information in organizational resilience journals and transformational entrepreneurship journals. We understand organizational resilience well, especially as it relates to organizations/companies, so organizational resilience has an ecosystem that shapes it. Be it human resources, environmental resources, financial resources, and the social-economic-community environment. Therefore, leaders and employees within an organization or company must possess transformational entrepreneurship behavior to achieve sustainable growth.

From the journal period reviewed for Transformational Entrepreneurship (TE) from 2005 to 2019 and Organizational Resilience (OR) from 1997 to 2020, we see a common thread through meta-analysis, where we find theoretical gaps that contribute to generating novelty in the form of conceptual propositions.

## Results

**Figure 2.**
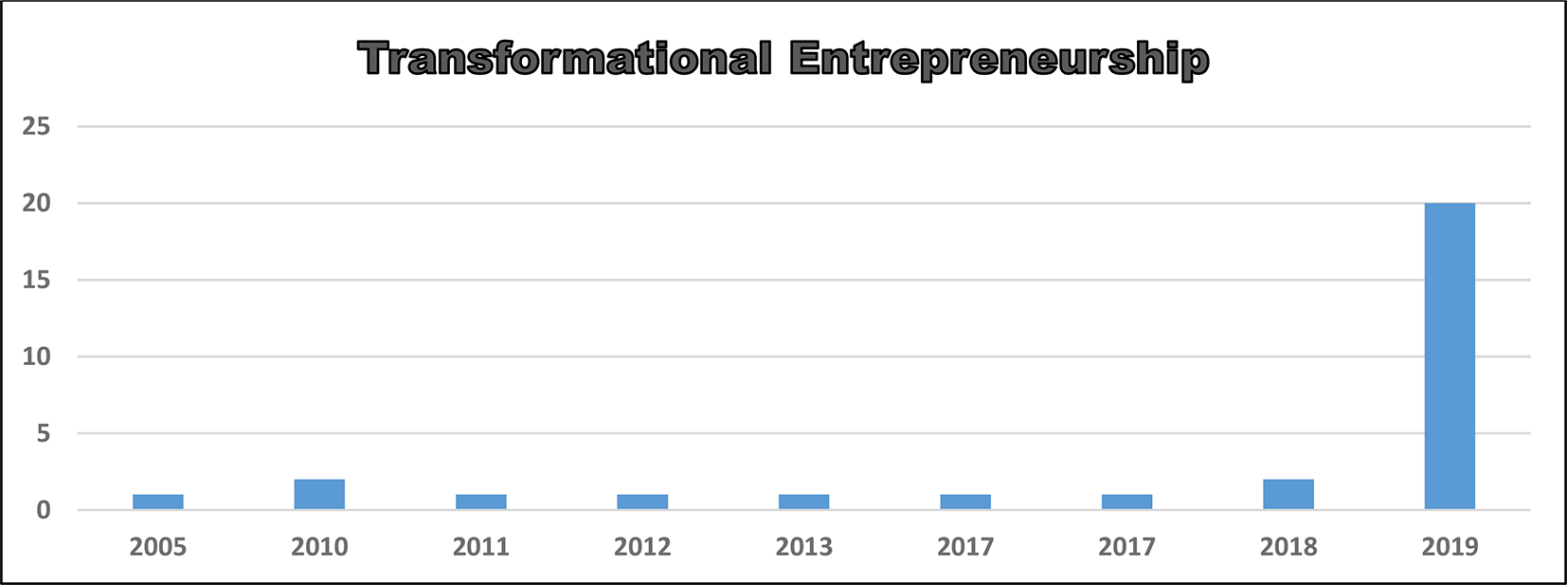
Transformational entrepreneurship (behavior): number of publications per year from 2005–2019.

**Figure 3.**
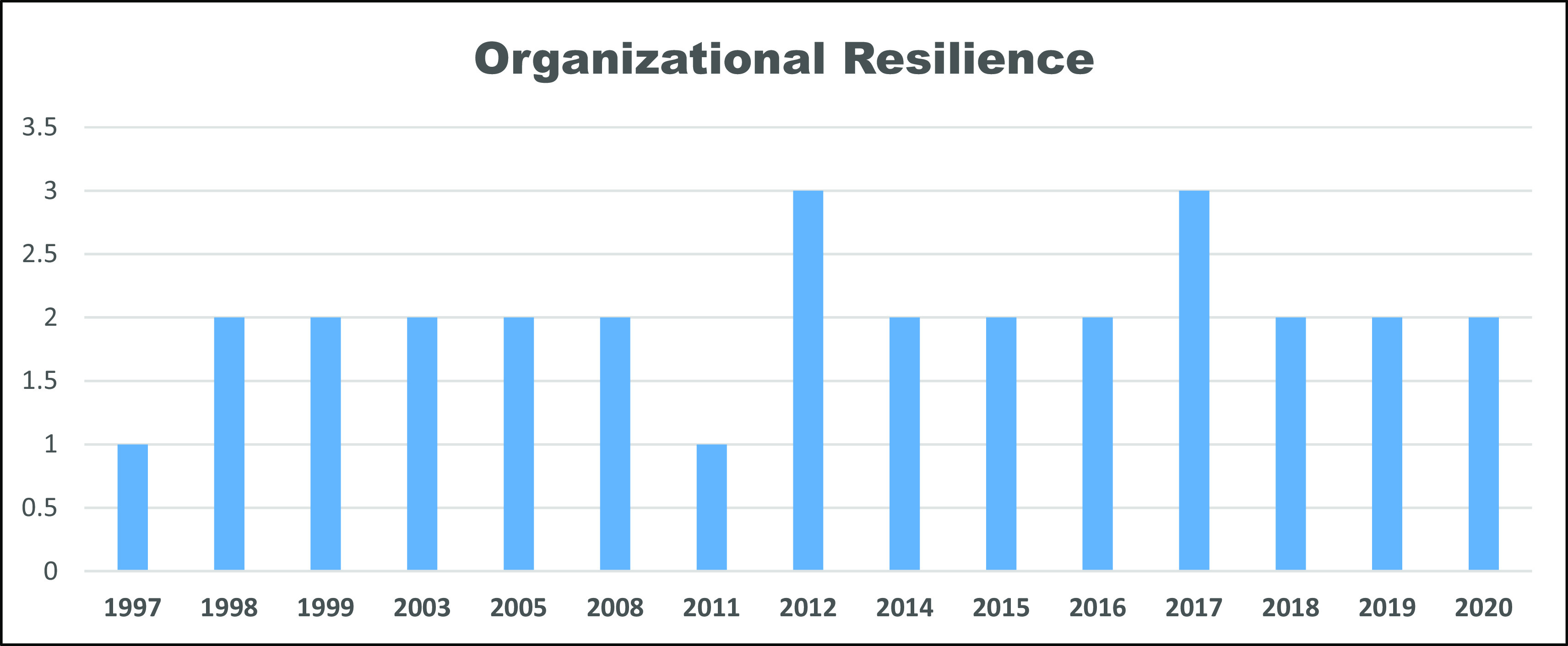
Organizational resilience: number of publications per year from 1997–2020.

Figures 2 and 3 explain the number of publications obtained per year and reveal the trend of increasing research using these variables or constructs, as well as building novelty and providing socio-economic and industrial impacts and contributions. The trend of increasing this research follows the increasing number of publications in the field of transformational entrepreneurship—transformational entrepreneurship behavior, starting in the 1990s and 2010 and continuing to grow and develop, respectively, in the last 10 years, and organizational resilience has fluctuated and tended to be stable in the last 25 years.

### Transformational entrepreneurship behavior determinant framework

The framework model ascertains the proposition of organizational transformational entrepreneurship behavior, which is determined by the organization’s resilience and the ecosystem in which the organization is located. How strongly organizational resilience is influenced by individual aspects within the organization, namely, the psychological capital of the top leadership of the organization and quality of the relationship between the top leadership of the organization and its subordinates ( Dansereau et al., 1975; Graen et al., 1982; Graen & Cashman, 1975; Hind et al., 1996; Horne, 1997; Kantur & İşeri-Say, 2012; Luthans et al., 2015; Mallak, 1998, 1999; Wilson & Ferch, 2005; Youssef, 2004), as well as by aspects of organizational aspects, namely the organization’s ability to explore and exploit simultaneously, and the organization’s competitive strategy ( Adler et al., 1999; Caspin-Wagner et al., 2012; Doe, 1994; Gibson & Birkinshaw, 2004; Horne, 1997; Iyer et al., 2006; Kantur & İşeri-Say, 2012; Katila & Ahuja, 2002; Mallak, 1998).

Meanwhile, the determinants of transformational entrepreneurship behavior from the internal side of the organization include the capability to explore and exploit opportunities simultaneously and in a balanced manner ( *ambidexterity)* ( Benner & Tushman, 2003; Gibson & Birkinshaw, 2004; O’Reilly & Tushman, 2008, 2013; Tamayo-Torres et al., 2017) according to transformational innovative and risk-taking behavior. Likewise, the internal determinants of TEB include a competitive advantage strategy because it must be owned by every organizational company ( Barney, 1991, 1995; Porter, 1989, 1996; Stonehouse & Snowdon, 2007), which follows transformational innovative and proactive behavior.

### Government regulation

Cull *et al.* (2013) argued that a need exists for joint efforts to measure environmental quality that impacts business growth and investment to provide benefits to socio-economic and environmental growth in a region. Therefore, the World Bank (2013) provides guidelines on the ease-of-doing-business index based on the complexity of business regulations and respect for protecting property rights. This index is measured to determine the ease of doing business, starting a business, including managing and obtaining permits, process and traveling time, including permits to build company areas or business buildings, ease of obtaining loans, and protection of investors, including permits to establish banks and manage money markets.

Ciccone and Papaioannou (2007) argued that the process of registering a new business entity for an existing business is significantly negatively related to new business investment. Meanwhile, Klapper, Laeven, and Rajan (2006) found that easier and more concise regulations will help capital inflows invest, including the growth of business activities in the building of industry. Therefore, the complexity of regulations determines the quality of the environment and space in conducting business, which significantly impacts economic growth ( Cull et al., 2013).

**Figure 4.**
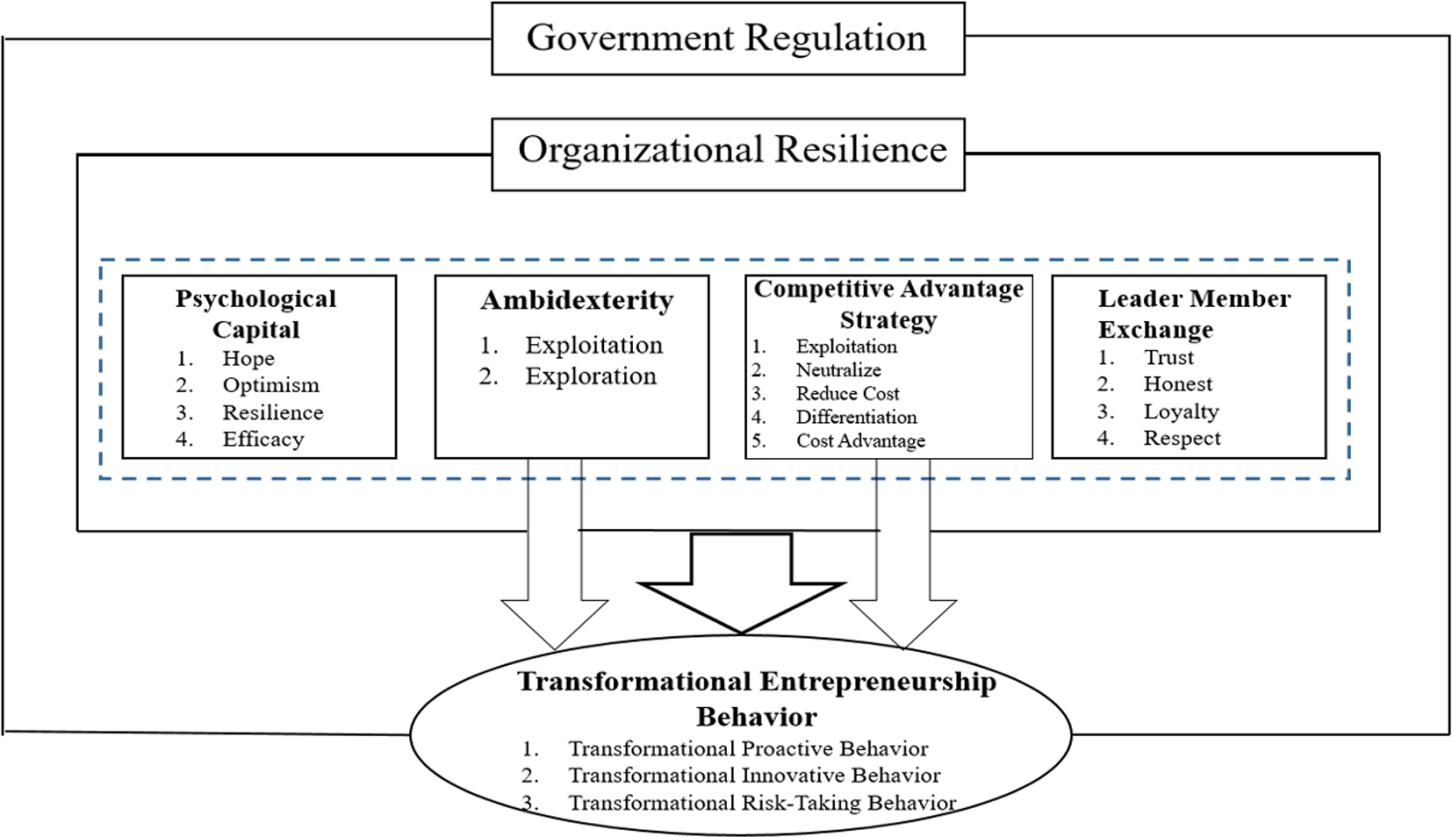
Review framework.

Referring to the research framework ( Figure 4), external factors, such as the environment, are the determining factors that influence companies, especially MSMEs, as well as microfinance institutions and multi-finance cooperatives. Therefore, the company’s orientation toward organizational resilience and transformational entrepreneurial behavior and the results obtained can certainly be influenced by external factors, namely the environment that is a sustainable supporter; in this case, government regulations play an extremely noteworthy role in succeeding the socio-growth of industry, business, and MSMEs, as well as microfinance institutions.

### Organizational resilience

A suitable increase in the resilience advantage is required to deal with unpredictable and uncertain catastrophe and loss conditions. Resilience includes organizational, individual, community, and regional resilience ( Kantur & İşeri-Say, 2012). Resilience in the real sense also includes all aspects of conducting assessments, including various disciplines ( Gunderson & Holling, 2002; Werner & Smith, 1977).

Organizational resilience capabilities are prepared and built by implementing crisis management and preparing human resource capabilities and organizational resources with high reliability so that they can anticipate and cope with sudden and disruptive disasters and environmental changes ( Sutcliffe & Vogus, 2003; Weick et al., 1999; Weick & Sutcliffe, 2001).

Moreover, Horne and Orr (1997) argued that resilience is the capacity and capability possessed by organizations, individuals, communities, and regions to prepare themselves for disasters and volatile conditions so that they can respond carefully and overcome these problems quickly. Similarly, Mallak (1998) held that an organization’s resilience can transform it to achieve sustainable socio-business and environmental growth.

Robb (2000) also stated that organizations with strong resilience can provide great performance following current organizational goals and continue to innovate and adapt to changing market conditions and technology so that organizations can grow and develop sustainably. Therefore, organizations with strong resilience automatically have good capabilities to avoid disturbances while also dealing with turbulence disturbances that occur effectively.

Kantur and İşeri-Say (2012) developed an organizational resilience framework and identified its determinants:

1. The perceptual stance describes the organization’s positive perception, especially that of top leaders. Individuals with positive perceptions have high psychological capital.
2. Contextual integrity describes employees’ involvement and empowerment. Employees with high involvement and empowerment have a quality relationship with the organization’s top leadership. With these quality relationships, employees gain the trust of the organization’s top leadership so that they get the support of resources that allow them to be more involved and empowered than others.
3. Strategic capacity describes an organization’s ability to deal with uncertain situations. This ability is reflected in the ability to conduct exploration and exploitation simultaneously, considering that this uncertain situation may present new opportunities that need to be explored further, but at the same time, existing opportunities need to be continuously exploited.
4. Strategic acting describes an organization’s creative, flexible, and proactive actions. These three actions are included in the organization’s competitive strategy to outperform its competitors. By acting creatively, flexibly, and proactively, organizations can offer products that are different from those in the market or offer similar products at more competitive prices. Therefore, it is necessary to build a competitive advantage, which is a decisive factor in the long-term success of a business. When a business can create a competitive advantage over its competitors, growth and revenue will increase ( Barney, 1991).

It is also important to have a competitive advantage based on the organizational culture built, strategic assets owned, and the business model applied to retain existing, loyal, and potential customers and serve the target market through a high-efficiency process ( Barney, 1991, 1995; Flamholtz & Randle, 2012; Porter, 1989, 1996; Stonehouse & Snowdon, 2007).

*Psychological capital*

The attitude of having a desirable perception represents a perception of the existing reality rooted in the wisdom to create a positive mindset and have high commitment. Building a positive perception and widespread dissemination within the organization will become a guideline and commitment of employees and the organization to strengthen the resilience of the organization. Meanwhile, understanding the existing reality will build a perception of self-image, which is an important element in building organizational resilience ( Hind et al., 1996).

According to Coutu (2002), the characteristics of strong organizational resilience are based on the meaning of real reality. This meaning builds a positive image so that the perception of the meaning of organizational resilience is to face bad and disturbing problems and situations ( Mallak, 1998). According to Flach (2004), optimism (optimism) and hope (hope) are important elements in building perceptions. This positive perception increases the resilience of an organization.

According to Flach (2004), optimism and hope are noteworthy elements of constructive perception. This positive perception increases organizational resilience, as needed in the perceptual stance element. The perceptual stance construct is compatible with psychological capital. Therefore, psychological capital (PsyCap) is a decisive factor that determines organizational resilience to support the success of transformational entrepreneurship behavior in increasing employee performance.

This aligns with Youssef (2004) and Luthans, Youssef, and Avolio (2015), who held that psychological capital (PsyCap: Psychological Capital), as it is now widely recognized human and social capital, is a take-off from economic capital, where existing resources are invested and utilized for future returns. Operationally, PsyCap can be explained as follows: (1) having high self-efficacy in making the decisions needed to succeed in ever-changing challenges; (2) creating a positive korsa spirit (optimism) regarding how to “build success now and in the future”; (3) tenacious, persistent and diligent in achieving the goal, and directing all power and ability to reach the path to the expected goal; and (4) when facing problems and challenges, being able to recover and then rise again through effective resilience to achieve success ( Luthans et al., 2004, 2015). Thus, in sum, PsyCap, individually and as an organization, is an important determinant of building organizational resilience.

*Leader-member exchange*

Employee engagement and empowerment are essential for employees to demonstrate appropriate behavior when faced with adversity or an unpredictable turbulent environment. Therefore, the unity of thought and action between leaders and employees is needed.

Facing great pressure from disasters, organizations must prepare themselves carefully, overcome these problems, and then adapt to changing environments and conditions. Mallak (1998) emphasized the importance of empowerment in the decision-making process from leaders to employees as an important element of organizational resilience. Empowering employees in decision-making enables them to produce creative solutions with increased authority and capability.

The leader–follower relationship is a mutually supportive interpersonal relationship. Mutual trust and respect are essential in an organization ( Dulebohn et al., 2012), and thus, understanding the distance at the point of a two-way relationship between “leaders and followers/subordinates” is highly important to increase positive outcomes for employees and organizations ( Dansereau et al., 1975; Graen et al., 1982; Graen & Cashman, 1975)”. According to Anand et al. (2018), the power distance factor between leaders and followers is important for improving communication and good relationships. Similarly, Antonakis and Atwater (2002) and Napier and Ferris (1993) posited that communication within the organization runs smoothly because of the good relationship between leaders and subordinates so that the goals and missions of achieving organizational performance are met.

According to Gouldner (1960), leadership-subordinate relationships must be rooted in reciprocal personal relationships that support each other, communicate with each other, and are open to each other. In building high-quality relationships, leaders provide empowerment, trust, and support in the form of resource allocation, improvement in the quality of key tasks and functions, and through coaching and mentoring ( Ilies et al., 2007; Maslyn & Uhl-Bien, 2001; Settoon et al., 1996). Therefore, to form high-quality relationships, a leadership role is needed to motivate, build relationships, and provide trust and empowerment to subordinates to ensure that they are expected to contribute to producing an effective performance for the organization ( Settoon et al., 1996; Wayne et al., 1997).

Furthermore, Weick (1993) posited that continuous open communication is an important factor in coordinating superiors and subordinates in an organization. Likewise, Horne and Orr (1997) recommended that to foster effective organizational resilience, leaders must communicate effectively about the company’s goals, mission, and vision to their employees to build trust—an important principle in building such resilience.

Van Breukelen, Schyns, and Le Blanc (2006) proposed an approach to building a good leader-member relationship process through open and honest two-way interactions. This confirms that LMX theory is a characteristic of a dynamic causal relationship between leaders and their subordinates while simultaneously fostering commitment to advancing the organization ( Northouse, 2012).

*Ambidexterity*

March (1991) identified two categories of implementation in an organization: (1) explorinh novel opportunities and findings because they are related to innovation, and (2) exploiting to develop and deepen existing resources, as well as to update and improve the quality and capabilities of organizational resources. Companies and organizations must “conduct exploration and exploitation simultaneously and in a balanced manner by utilizing available resources and good managerial capabilities to obtain better organizational performance ( Chandrasekaran et al., 2012; Kristal et al., 2010; Salvador et al., 2014).

Organizations or companies sometimes face an unstable and hostile environment requiring organizations to engage in “exploration and exploitation, thus becoming an ambidextrous organization ( Benner & Tushman, 2003; Lubatkin et al., 2006; Raisch & Birkinshaw, 2008; Tamayo-Torres et al., 2014, 2017).” Therefore, ‘ambidexterity’ is a company’s capability to carry out exploration and exploitation activities simultaneously and in a balanced manner ( O’Reilly & Tushman, 2004).

Organizations need to maintain an appropriate combination of exploration and exploitation—a balance between the organization’s ability to exploit and explore existing and new opportunities—to produce optimal company performance ( Cao et al., 2009; Lubatkin et al., 2006). This is according to O’Reilly and Tushman (2004), who highlighted the need for simultaneous execution of exploration and exploitation to achieve a balance that results in the survival and success of the organization or company. Therefore, combining exploration and exploitation is important to achieve improved long-term organizational performance ( Benner & Tushman, 2003; Gibson & Birkinshaw, 2004; Lubatkin et al., 2006; Tamayo-Torres et al., 2017). In essence, organizations that conduct exploration and exploitation activities simultaneously and in a balanced manner, can deal with situations and environmental conditions that change dramatically. This aids organizations to receive new opportunities as an organization with effective resilience capabilities ( Mallak, 1998).

Therefore, companies must take advantage of existing opportunities and customers and seek new opportunities and customers with a proactive, creative, and innovative approach in a balanced and concurrent way (ambidexterity; ( Benner & Tushman, 2003; Cao et al., 2009; Gibson & Birkinshaw, 2004; Lubatkin et al., 2006; O’Reilly & Tushman, 2008, 2013; Tamayo-Torres et al., 2017).

Furthermore, the company uses the ability to act proactively, creatively, and innovatively by exploiting existing markets/opportunities while simultaneously developing creative and innovative behavior by exploring new opportunities by daring to take risks. This is in line with transformational innovative behavior and proactive behavior that such behaviors aim to change and transform.

*Competitive advantage strategy*

The capability of the organization to prepare itself by anticipating potential disasters and crises must be followed by strategic organizational capacity and strategic actions, where the behavior of employees in the organization must be creative, agile, sensitive, resilient, and proactively oriented toward innovation-resulting solutions ( Duchek, 2020; Kantur & İşeri-Say, 2012; Wenzel et al., 2021).

Competitive advantage strategy has proven to be an extremely important element of how organizations grow and develop to achieve their goals and objectives ( Ehie & Muogboh, 2016; Kharub & Sharma, 2016, 2017; Korutaro Nkundabanyanga et al., 2014; Olhager & Feldmann, 2018; Stonehouse & Snowdon, 2007). Based on a literature review, the competitive advantage strategy comprises the following four categories: (1) These manuscripts describe the concept of competitive strategy ( Kathuria et al., 2007; Porter, 1985, 2000; Skinner, 1969, 1985). (2) These studies define competitive advantage strategies ( Dangayach & Deshmukh, 2006; Ward & Duray, 2000). (3) The literature correlates the direct effect of competitive advantage strategy and firm performance ( Amoako-Gyampah & Acquaah, 2008; Olhager & Feldmann, 2018). (4) These studies emphasize the development of resource capabilities to support a company’s competitive advantage strategy ( Jarzabkowski et al., 2016; Sharma & Kharub, 2014).

Organizations must build a competitive advantage to develop a business focusing on its target market. Meanwhile, in business, a competitive advantage category is needed, according to Barney (1991), VRIN: Valuable, Rare, Inimitable, and Non-Substitution. If this competitive advantage is built properly, the continuity of the organization or company will continue in the long term ( Jacobsen, 1988; Porter, 1985). According to Porter (1989, 1995), Barney (1991, 1995), Christensen (2006), Sigalas, Economous, and Georgopoulos (2013), Stonehouse and Snowdon (2007), and Nkundabanyanga, et al. (2014).

Meanwhile, organizations that face environmental pressures with dynamics at an uncertain level of turbulence must be able to act creatively, flexibly, and proactively for the emergence of solution-oriented and elastic organizational behavior to ensure that they can compete ( Kantur & İşeri-Say, 2012). These three actions are included in the organization’s competitive strategy to outperform its competitors. By acting creatively, flexibly, and proactively, organizations can offer products that are different from those in the market or offer similar products at more competitive prices.

To compete and win against competitors, the company must have a competitive advantage strategy by encouraging different advantages, such as price advantages focusing on the target market, advantages of the network owned, advantages of having resources, and advantages of good name/reputation. This will encourage the company to act creatively and innovate continuously, ahead of similar competitors and dare to take risks ( Korutaro Nkundabanyanga et al., 2014; Sigalas et al., 2013; Stonehouse & Snowdon, 2007). This is in line with transformational entrepreneurship behavior that always transforms by daring to take risks, act proactively and innovate continuously.

Ultimately, Doe (1994) emphasized that organizations with high resilience capabilities must be supported by resource capabilities, including good human resources. This will help them absorb crises and disasters resulting in drastic changes and view change as an opportunity that must be seized because it benefits the organization as a whole.

The characteristics of organizations with high resilience include timely and appropriate responses and the capacity for creative renewal ( Kantur & İşeri-Say, 2012). That is, the response consists of proactive, innovative, and risk-taking behaviors ( Kantur & İşeri-Say, 2012). Meanwhile, Hunter (2006) argued that resilient individuals who have high resilience can change crises by carrying out organizational transformation through transformational entrepreneurship behavior ( Maas et al., 2019; Maas & Jones, 2017) to generate individual and organizational growth on an ongoing basis.

### Transformational entrepreneurship behavior

Change-oriented organizational leaders invariably anticipate sudden changes that disrupt environmental situations and conditions to continue bringing the organization to improve its performance ( Lafley, 2009). This leader must bring the organization to an entrepreneurial spirit by innovating, acting proactively, and taking risks ( Covin & Slevin, 1991; Miller & Friesen, 1982). Bass (1985) and Howell and Avolio (1993) argued that transformational leadership is related to innovation in organizations. Likewise, Crant (2000) and Deluga (1998) found that transformational leaders are associated with transformational behavior, including transformational behavior that is proactive, innovative, and willing to take risks.” In other words, transformational leadership has been known to be associated with entrepreneurship.

Therefore, a need exists to renew thinking to stimulate entrepreneurship to support socio-economic growth, including community development. In this context, “transformational entrepreneurship” is comprehensive and holistic in promoting entrepreneurship, including socio-economic development and community development, so that they can interact and collaborate synergistically to create positive opportunities and benefits and reach a wider scale ( Maas et al., 2019).

Entrepreneurship plays an extremely important role in encouraging sustainable socio-economic development. It needs to be studied further to determine whether the organization has a capable capability, adequate capacity, and a supportive ecosystem so that the right policies are needed to build socio-economic growth, including community development, in an optimal balance. As mentioned above, the key to success in dealing with volatile situations and conditions with high uncertainty is not just survival but sustainability. Therefore, companies do not need only economic benefits (short-term) but also social impacts (long-term) to ensure that the company’s success increases societal welfare ( Maas et al., 2019; Maas & Jones, 2017; Xu & Maas, 2019). According to Marmer (2012), entrepreneurship can transform institutions so that they are profit-oriented (e.g., companies) to incorporate a social perspective into their goals.

The author argues that it is necessary to develop propositions to empirically prove the model review framework, as presented in Figure 4; this can be applied to companies in all industries, including microfinance institutions, multi-finance cooperatives, and MSMEs to create socio-economic growth and build the sustainability of the company itself, in addition to environmental and community development ( Maas et al., 2019; Xu & Maas, 2019). This transformation of entrepreneurship behavior is needed in all organizations or companies for a sustainable existence. Therefore, this idea is needed, which is obtained through case studies of the entrepreneurship industry via the three main categories of organizational resilience, ambidexterity, and competitive advantage strategy ( Kantur & İşeri-Say, 2012; Korutaro Nkundabanyanga et al., 2014; Lubatkin et al., 2006; Sigalas et al., 2013).

Through an in-depth study of the bibliography, the author realizes that the conceptual paper that builds a framework review model is still limited. The author supports that TEB can be applied to all companies and industries, including microfinance institutions, multi-finance cooperatives, and MSMEs. However, adopting a different approach is necessary, and examining the determinant factors and ecosystems supporting TEB formation may be required.

## Discussion

The purpose of this in-depth study, review of the literature, and collection of research questions is collating all conceptual approaches to TEB and organizational resilience in companies and various industries to promote socio-economic growth and achieve environmental sustainability and balanced community development. Furthermore, an in-depth systematic review yielded a model framework ( Figure 4). Based on the model, this framework is subsequently analyzed to identify research gaps and offer avenues for further research.

This literature review clearly describes the ecosystem that drives the development of transformational entrepreneurial behavior ( Xu & Maas, 2019) and the determinants of organizational resilience ( Kantur & İşeri-Say, 2012). However, research on transformational entrepreneurship behavior for a company can predominantly be conducted in the context of the private sector, state-owned companies, MSMEs, microfinance institutions, and multi-finance cooperatives. Therefore, the articles in the literature review regarding organizational resilience and its influence on transformational entrepreneurial behavior are still conceptual and represent a theoretical gap with respect to producing a basic framework or model.

The author attempts to link the research flow of organizational resilience and transformational entrepreneurial behavior more closely; Mass and Jones (2017) recommended conducting empirical research to be applied by companies in various industries to prove conceptual thinking. Likewise, they argued for considering the different determinants and ecosystems that shape them concerning transformational entrepreneurship behavior.

Referring to the construct that forms transformational entrepreneurial behavior ( Maas et al., 2019), the existence of an ecosystem, namely, factors outside the company that shape it ( Xu & Maas, 2019). The importance of TEB in encouraging socio-economic growth does not stand alone ( Maas et al., 2019; Xu & Maas, 2019). It is necessary to provide an ecosystem that supports infrastructures, policies/regulations, culture (from the parent company/coach, government, environment, and society), markets, and finance/funding ( Isenberg, 2011; Mason & Brown, 2014; Oh et al., 2016; Smith, 2006; Xu & Maas, 2019) to ensure that the climate of innovation and entrepreneurship is formed. Ecosystems that include infrastructure, policies/regulations, culture, parent company support, the environment, and society significantly contribute in the literature review, particularly in building transformational entrepreneurship behavior ( Xu & Maas, 2019).

Furthermore, the author argues that the developed model framework can be implemented using quantitative studies through theory development, which is a theoretical gap in line with the definition of organizational resilience and transformational entrepreneurship behavior. Thus, the adaptation of constructs that shape organizational resilience and the determinants of transformational entrepreneurship behavior can be applied to companies in various industries.

### Limitations

This conceptual study, created through a literature review, explains the determinants of organizational resilience and transformational entrepreneurship behavior, but it has several limitations in this case. The first limitation is that the literature review focuses on academic journals located in Scopus, hosted by EBSCO and Web of Science (WoS) databases, both conceptual and empirical. Likewise, Podsakoff *et al.* (2005) argued that it is necessary to conduct an intensive literature review and improve the quality of the literature review and the findings obtained to ensure that a holistic picture is obtained. The next limitation is that the information obtained from the literature review is sometimes inadequate; therefore, the model of the review framework needs to be further developed to obtain a different view of other potential ecosystems.

The author refers to Pawson (2006) in selecting a paper that must follow the intended focus of its suitability and purpose. Indubitably, how the influence of organizational resilience on transformational entrepreneurship behavior can be applied in companies of all industries, especially for MSMEs and microfinance institutions, and multi-finance cooperatives. Furthermore, the limited quality of the information obtained from the literature review prompted us to develop only a probable theoretical framework.

## Conclusion

The stream of research that focuses on TEB is still rare. Currently, this research is increasing, but more so on a conceptual level. Thus far, both conceptual and empirical research has focused more on entrepreneurship behavior that aims to achieve economic gain. Therefore, the literature review in this conceptual paper aims to produce research paths for the present and the future, including the determinants and ecosystems that shape TEB, and to generate broad insights that can be implemented in contemporary companies of all industries. Furthermore, this study also focuses on the effect of organizational resilience on transformational entrepreneurship behavior and the determinant factors shaping organizational resilience, rooted in an in-depth literature review.

The author conducts a careful review of the literature to identify the theoretical gaps that have been vague and then combines two streams of research—namely, organizational resilience and transformational entrepreneurship behavior—in building a model framework. Therefore, we developed an integrative review framework ( Figure 4) as an empirical research model. Finally, numerous future research opportunities exist to develop other review framework models previously developed by scholars.

## Data Availability

No data are associated with this article. Figshare: PRISMA and PRISMA abstract checklists and flow chart for ‘How to link organizational resilience to transformational entrepreneurship behavior as theoretical framework gap’. https://doi.org/10.6084/m9.figshare.22591693.v1. ( Gunawan, 2023). Data are available under the terms of the Creative Commons Attribution 4.0 International license (CC-BY 4.0).
