# Supplementary material for: How to link organizational resilience to transformational entrepreneurship behavior as theoretical framework gap – A systematic literature review
Source: F1000Res. 2023 Jun 28;12:761. [Version 1] doi: 10.12688/f1000research.133459.1 (PMC10333783; doi:10.12688/f1000research.133459.1)
Supplement: Peer review history

# Peer review: Reviewer response for version 1

**DOI:** 10.5256/f1000research.146450.r183920

**Published:** 2023-07-10

**Authors:** Odafe Egere

**Affiliations:** 1 Entrepreneurship & Innovation, Department of Marketing, Innovation, Strategy & Operations (MISO), University of Leicester, Leicester, England, UK

The systematic review provided a well structured insight into understanding TEB considering OR. The background and rationale is well presented. The method and analysis is well detailed and would help future research. The review would help further research exploring TEB and the propositions discussed would inform additional layers of research, which is focus in deepening this knowledge area. An effective conclusion is discussed and provided the review contribution and future research opportunities.

Are the rationale for, and objectives of, the Systematic Review clearly stated?

Yes

Is the statistical analysis and its interpretation appropriate?

Not applicable

Are sufficient details of the methods and analysis provided to allow replication by others?

Yes

Are the conclusions drawn adequately supported by the results presented in the review?

Yes

Reviewer Expertise:

Transformational entrepreneurship and entrepreneurial development.

I confirm that I have read this submission and believe that I have an appropriate level of expertise to confirm that it is of an acceptable scientific standard.

# Peer review: Reviewer response for version 1

**DOI:** 10.5256/f1000research.146450.r183921

**Published:** 2023-07-10

**Authors:** Gideon Maas

**Affiliations:** 1 International Centre for Transformational Entrepreneurship, Coventry University, Coventry, UK

This is a systematic literature review that attempts to explore transformational entrepreneurship behavior, socio-economic growth and sustainability, community development, and organizational resilience. The authors explained the systematic literature review process they followed in detail. They provided limitations of this research which include the limited number of research articles. As the research grows over time, more detailed and deeper discussions would be possible. 

 The article can do with another round of light touch editing.

Are the rationale for, and objectives of, the Systematic Review clearly stated?

Yes

Is the statistical analysis and its interpretation appropriate?

Not applicable

Are sufficient details of the methods and analysis provided to allow replication by others?

Yes

Are the conclusions drawn adequately supported by the results presented in the review?

Yes

Reviewer Expertise:

Entrepreneurship.

I confirm that I have read this submission and believe that I have an appropriate level of expertise to confirm that it is of an acceptable scientific standard.

# Peer review: Reviewer response for version 1

**DOI:** 10.5256/f1000research.146450.r183922

**Published:** 2023-07-10

**Authors:** Andreas Walmsley

**Affiliations:** 1 Marjon Business, Plymouth Marjon University, Plymouth, England, UK

The paper uses a systematic literature review to explore the relationship between organisational resilience and transformational entrepreneurship. The focus on transformational entrepreneurship in particular is welcome; it is an important concept with an increased amount of research being afforded its investigation (as also demonstrated in the paper, Figure 2). The study, based on 22 included articles that were reviewed, develops a theoretical framework whereby a number of factors of the ecosystem are presented that influence transformational entrepreneurship behaviour outcomes. The four categories of factors that together offer organisational resilience are: psychological capital, ambidexterity, competitive strategy and leader/member exchange. All of this takes place within a broader government regulatory framework. The study thereby also taps into a stream of literature that seeks to better understand the role of context in shaping entrepreneurship. Results should be of interest to those interested in transformational entrepreneurship specifically, but also the wider community of enterprise/entrepreneurship scholars.

Are the rationale for, and objectives of, the Systematic Review clearly stated?

Yes

Is the statistical analysis and its interpretation appropriate?

Not applicable

Are sufficient details of the methods and analysis provided to allow replication by others?

Yes

Are the conclusions drawn adequately supported by the results presented in the review?

Yes

Reviewer Expertise:

Entrepreneurship

I confirm that I have read this submission and believe that I have an appropriate level of expertise to confirm that it is of an acceptable scientific standard.
